# Tetrandrine Prevents Bone Loss in Ovariectomized Mice by Inhibiting RANKL-Induced Osteoclastogenesis

**DOI:** 10.3389/fphar.2019.01530

**Published:** 2020-01-10

**Authors:** Zeyuan Zhong, Zhi Qian, Xu Zhang, Fancheng Chen, Shuo Ni, Zhanrong Kang, Fangxue Zhang, Dejian Li, Baoqing Yu

**Affiliations:** ^1^ Department of Orthopedics, Shanghai Pudong Hospital, Fudan University Pudong Medical Center, Shanghai, China; ^2^ Shanghai Medical College, Fudan University, Shanghai, China

**Keywords:** postmenopausal osteoporosis, tetrandrine, osteoclasts, differentiation, OVX mice

## Abstract

Postmenopausal osteoporosis (PMOP) is a metabolic bone disease characterized by decreased bone density and strength due to the imbalance between osteogenesis and osteoclastogenesis. Postmenopausal estrogen withdrawal increases proinflammatory cytokines and increases the serum level of Receptor activator of NF-kB ligand (RANKL)/Osteoprotegerin (OPG), which then leads to the overactivation of osteoclastogenesis. Tetrandrine, a bis-benzylisoquinoline alkaloid, has been widely used in the treatment of rheumatoid arthritis clinically in China. Here, we demonstrate that tetrandrine significantly prevented ovariectomy-induced bone loss and inhibited RANKL-induced osteoclastogenesis. *In vivo*, we found that intraperitoneal injection of tetrandrine (30 mg/kg) every other day markedly reduced bone loss in ovariectomized mice and the serum levels of TRAcp5b, TNF-a, IL-6, CTX-I, and RANKL/OPG were significantly decreased. *In vitro*, we found that tetrandrine significantly inhibited osteoclast differentiation in bone marrow monocytes (BMMs) and RAW264.7 cells according to the results of osteoclastogenesis‐related gene expression, tartrate-resistant acid phosphatase (TRAP) staining and actin-ring formation as well as bone resorption assay. Mechanistically, tetrandrine inhibited RANKL‐induced osteoclastogenesis by suppressing NF-kB, Ca2^+^, PI3K/AKT, and MAPKs signaling pathways. Taken together, our findings suggest that tetrandrine suppresses osteoclastogenesis through modulation of multiple pathways and has potential value as a therapeutic agent for PMOP, especially for those suffering from RA and PMOP at the same time.

## Introduction

Postmenopausal osteoporosis is the most common type of primary osteoporosis, and, resulted from global aging, its incidence is increasing recently (Compston et al., 2019). PMOP leads to an increased risk of fractures and chronic pain, which reduces the quality of life for older women and increases the burden on society (Gartlehner et al., 2017). After menopause, estrogen withdrawal weakens its inhibitory effect on inflammatory cytokines, leading to the increase of inflammatory cytokines in the body, which further breaks the balance between osteoblast‐mediated bone formation and osteoclast‐mediated bone resorption (Eastell et al., 2016). Osteoclast is a kind of multinucleated giant cells, which differentiated from hematopoietic precursor cells of monocyte-macrophage lineage and had the function of bone resorption (Boyle et al., 2003). Osteoclastogenesis is a multi‐stage process regulated by numerous factors, while macrophage colony‐stimulating factor (M‐CSF) and receptor activator of nuclear factor‐kappa B (NF‐κB) ligand (RANKL) are the key cytokines. The cytokine M‐CSF is a prerequisite for the induction of osteoclast. It facilitated the proliferation and survival of osteoclast precursors and made it possible for RANKL to bind to RANK on the cell membrane. (Shinohara et al., 2008; Cao, 2018; Liu et al., 2018)The binding of RANKL to the RANK receptor activated multiple intracellular signal pathways, including NF-kB, MAPKs, Ca^2+^, and PI3K/AKT pathways, and thereby initiates osteoclast differentiation and bone resorption by inducing transcription and expression of osteoclast associated genes, such as tartrate-resistant acid phosphatase (TRAP), cathepsin K (CTSK), c-Fos, and NFATc1 (Nemeth et al., 2011; Sobacchi et al., 2013; Croft et al., 2017). Therefore, it is a feasible strategy to inhibit osteoclastognenesis by suppressing the downstream pathway activated by RANKL to prevent and treat PMOP. Tetrandrine, a bis-benzylisoquinoline alkaloid, has been isolated from Stephania tetrandra S Moore, and other Chinese herbs. Previous studies have demonstrated that tetrandrine has anti-inflammatory, antineoplastic, immunologic, and antiallergenic effects, and it has been widely used clinically in the treatment of rheumatoid arthritis (Wang et al., 2004; Liu et al., 2017; Yu et al., 2018). PMOP has a strong correlation with rheumatoid arthritis considering inflammatory cytokines played key roles in both of them. Meanwhile, a large part of PMOP patients suffered from rheumatoid arthritis at the same time (Sapir-Koren and Livshits, 2017). As tetrandrine was proved to be effective in treating rheumatoid arthritis due to its anti-inflammatory effects (Jia et al., 2019), we hypothesized that tetrandrine could suppress osteoclastognenesis and serve as a promising candidate for anti-osteoporosis drug development. We carried out this study to investigate the effects of tetrandrine on ovariectomy-induced bone loss and to explore the possible underlying molecular mechanisms.

## Materials and Methods

### Reagents and Antibodies

Tetrandrine (purity >98%) was purchased from MedChemExpress (New Jersey, USA) and was dissolved in DMSO at a 10 mmol/L stock solution and stored -20℃. Further dilution was carried out in culture medium for cells and PBS medium for animals. Primary antibodies against CTSK, CTR, MMP-9, TRAF6, TRAP, GAPDH, and β-actin were purchased from Proteintech (Wuhan, Hubei, China). Primary antibodies against NFATc1, P-PI3K, AKT, P-AKT, P50, P-P50, P65, P-P65, IκBα, P-IκBα, ERK1/2, P-ERK1/2, JNK, P-JNK, P38, and P-P38 were obtained from Cell Signaling Technologies (Beverly, MA, USA). Primary antibodies against RANKL, OPG, and the ELISA kit of RANKL were obtained from ABclonal (Wuhan, Hubei, China). The ELISA kits of OPG, IL-6, TNF-α, TRAcp5B, and CTX-I were purchased from Sangon (Shanghai, China). A CCK-8 assay kit was purchased from Dojindo (Tokyo, Japan). A leukocyte acid phosphatase staining kit was obtained from Sigma‐Aldrich (MO, USA). Recombinant M‐CSF and Recombinant m-RANKL were obtained from R&D Systems (Minneapolis, MN, USA). The cell culture medium that alpha‐modified minimal essential medium (α‐MEM), fetal bovine serum (FBS), and penicillin-streptomycin were purchased from Thermo Fisher Scientific (Scoresby, Vic., Australia).

### Ethics Statement

The study was approved by the Animal Ethics Committee of Shanghai Pudong Hospital. All surgical interventions, treatments and post‐operative animal care procedures were performed in accordance with the National Institutes of Health (NIH) Guide for the Care and Use of Laboratory Animals.

### Cell Culture

Bone marrow monocytes (BMMs) were isolated from the femoral bone marrow of 6-week-old C57BL/6 mice, which were euthanized according to the procedures approved by the Animal Ethics Committee of Shanghai Pudong Hospital. The extracted cells were transferred into a 75 cm^2^ culture flask with α‐MEM (adding 10% FBS, a 1% antibiotic mixture of penicillin and streptomycin) and incubated in an atmosphere of 5% CO2 at 37°C. Nonadherent cells were collected 12 h later and were incubated into another culture flask with α‐MEM (adding 10% FBS, a 1% antibiotic mixture of penicillin and streptomycin, 20 ng/ml M-CSF). BMMs from passages one to three were used in this study. Bone marrow stromal cells (BMSCs) adhered in 12 h and the adherent cells in the first flask were then cultured with α‐MEM (adding 10% FBS, a 1% antibiotic mixture of penicillin and streptomycin) to obtain BMSCs. The culture medium was changed every 2 days. BMSCs from passages one to five were used in this study. RAW264.7 cells were cultured with with α‐MEM containing 10% FBS. The culture medium was changed every 2 days. Passage the cells when 80%–90% confluence was attained.

### Cell Viability Assay

A CCK-8(Cell Counting Kit-8) assay was used to determined cell viability following the manufacturer’s protocol. RAW264.7 cells were seeded into 96‐well plates (5×10^3^ cell/well) and incubated with various concentrations of tetrandrine (0.25, 0.5, 1.0, 2.0, and 4.0 μM). After 24, 48, and 96 h of treatment, the cells were washed with PBS and 100 μl non‐FBS medium (α‐MEM) containing 10 μL CCK-8 solution was added to each well, then incubated the plate in the incubator with 5% CO2 at 37°C for an hour. Finally, measure the absorbance of each well at 450 nm by a micro‐plate reader. The same method was used to detect the cell viability of BMM cells with M-CSF (20 ng/ml).

### *In Vitro* Osteoclastogenesis Assay

BMMs (3 × 10^4^ cell/well) and RAW264.7 cells (5×10^3^ cells/well) were cultured on 96-well plates and divided into a control group and 5 groups treated with tetrandrine (0, 0.125, 0.25, 0.5, or 1 μM). For BMMs, the drug-treated groups were induced by M-CSF (20 ng/ml) for 3 days, then treated with M-CSF (20 ng/ml) and RANKL (50 ng/ml) for another 5 days. For RAW264.7 cells, the drug-treated groups were induced by RANKL (50 ng/ml) and M-CSF (20 ng/ml) for 5 days while the control groups were treated with M-CSF (20 ng/ml) only. The BMMs and RAW264.7 cells of the control group and the drug-treated groups were stained by tartrate-resistant acid phosphatase (TRAP) using a TRAP staining kit (Sigma-Aldrich, St. Louis, MO, USA) according to the manufacturer’s protocol. More than 3 nucleuses cells were regarded as osteoclast cells and counted for BMMs cells while more than 4 nucleuses for RAW264.7 cells. All the experiments were carried out three times.

### Actin Ring Formation Assay

BMMs were seeded into 96‐well plates and treated with different concentrations of tetrandrine in the presence of 20 ng/ml M‐CSF for 3 days and then treated with 20 ng/ml M‐CSF and 50 ng/ml RANKL for 5 days, the cells were fixed by paraformaldehyde (4%) for 15 min at room temperature. After being washed with PBS three times, cells were permeabilized with 0.3% Triton X‐100 for 5 min and blocked with 3% BSA in PBS. Stain the F‐actin rings with rhodamine‐conjugated phalloidin (Eugene, OR, USA) and the cell nuclei with DAPI. Then, capture the images by confocal laser scanning microscopy (Nikon, Tokyo, Japan). The number of multinucleated cells (>3 nuclei) and the number of nuclei were calculated.

### Resorption Pit Assay

A resorption pit assay was used to evaluate osteoclast function. BMMs were seeded into 6‐well plates at a density of 1 × 10^5^ cell/well and stimulated with 20 ng/ml M‐CSF for 3 days and then treated with 20 ng/ml M‐CSF and 50 ng/ml RANKL for 5 days until mature osteoclasts formed. Detached the Cells from the wells using a cell dissociation solution (Sigma, St. Louis, MO, USA) and then plated into 48‐well plates with bone slices. The mature osteoclasts were treated with different concentrations of tetrandrine in the presence of M‐CSF (20 ng/ml) and RANKL (50 ng/ml). After 48 h, bone slices were stained with Toluidine Blue to detect resorption pits. Use Image J software (NIH, Bethesda, MD, USA) to analyze the percentage of resorption areas of bone slices.

### Immunofluorescence Staining

An immunofluorescence staining was used to determine the effects of tetrandrine on the nuclear translocation of P65. The control group and drug-treated BMMs cells were fixed with 4% paraformaldehyde for 15 min. Then permeabilized the cells with 0.3% Triton X‐100 for 5 min and blocked with 3% BSA in PBS. The cells were incubated with anti-P65 antibody followed by biotinylated goat anti-mouse IgG antibody and fluorescein-conjugated streptavidin (Vector Laboratories, CA, USA). Cells were counterstained with propidium iodide.

### Ca^2+^ Concentration Detection

A fluo-4, AM kit (Solarbio, Beijing, China) was used to detect the Ca^2+^ concentration. Before the detection, we cultured BMMs with or without tetrandrine (1 µM) and RANKL (50 ng/ml) and M-CSF (20 ng/ml) for 48 h. Firstly, Add Pluronic F127 to Fluo-4, AM/DMSO solution and dilute it with HBSS. Secondly, culture BMMs with the solution for 20 min, then add in HBSS containing 1% FBS. After 40 min, wash the cells with HEPES buffer saline for 3 times and suspend the cells at a density of 1*10^5 cells/ml. The intracellular free calcium was detected at 494 nm by a flow cytometry (BD, New York, US). Then, the results were analyzed by FlowJo. Mean fluorescence intensity was used to evaluated the extent of Ca^2^ efflux.

### RT‐PCR

Quantitative real‐time polymerase chain reaction (qRT‐PCR) was used to quantify the mRNA expression of c-Fos, TRAcP, CTSK, and NFATc1. The total RNA of RAW264.7 cells treated with or without different concentrations of tetrandrine in the presence of RANKL (50 ng/ml) were extracted in 6‐well plates using TRIzol reagent (ThermoFisher Scientific, Scoresby, Australia). Next, 1000 ng of total RNA was reverse transcribed to synthesize cDNA using an RT‐PCR kit (Invitrogen, Carlsbad, CA, USA). The parameters of RT‐PCR were 5 min at 94°C, followed by 30 cycles of 40 sec at 94°C, 40 sec at 60°C, and 40 sec at 72°C, and then a final extension step of 5 min at 72°C. The reaction was performed using a ViiA™ 7 Real‐time PCR machine (Applied Biosystems, Paisley, UK). The cycle threshold (Ct) values were collected and normalized to the level of GAPDH. Data were analyzed using the 2-ΔΔCT method. The specific primers used are listed in **Table 1**.

**Table 1:** Primer sequences used in RT-PCR.

Gene	Forward primer	Reverse primer
NFATc1	5′‐CCAGCTTTCCAGTCCCTTCC‐3′	5′‐ACTGTAGTGTTCTTCCTCGGC‐3′
CTSK	5′‐TAGCACCCTTAGTCTTCCGC‐3′	5′‐CTTGAACACCCACATCCTGC‐3′
TRAcP	5′‐TGGGTGACCTGGGATGGATT‐3′	5′‐AGCCACAAATCTCAGGGTGG‐3′
c‐Fos	5′‐TTTCAACGCCGACTACGAGG‐3′	5′‐GCGCAAAAGTCCTGTGTGTT‐3′
GAPDH	5′‐AGGAGAGTGTTTCCTCGTCC‐3′	5′‐TGAGGTCAATGAAGGGGTCG‐3′

### Western Blotting

Western blotting was used to evaluate the effects of tetrandrine on NF-kB, MAPK, and PI3K/AKT pathways in BMMs. The BMMs were seeded (2×10^6^ cells/well) into 6-well plates and all 8 wells were divided into 2 groups, one group was treated with RANKL (50 ng/ml) and M-CSF (20 ng/ml) while another treated with RANKL (50 ng/ml), M-CSF(20 ng/ml), and tetrandrine(1 μM). Cells were evaluated by Western blotting at 0, 15, 30, and 60 min to observe phosphorylation of IκB, P65, P50, PI3K, AKT, ERK1/2, JNK, and P38. Western blotting was also used to measure the expression levels of osteoclastogenesis-related proteins, including MMP-9, TRAP, cathepsin K, CTR9, and NFATc1. RAW264.7 cells (control, treated with RANKL 50 ng/ml and tetrandrine 0 to 1 μM) were seeded in 6-well plates (6 × 10^5^ cells/well). After 5 days, the proteins were collected and measured by Western blotting. BMSCs were seeded in 6-well plates at a density of 2 × 10^5^ cells/well. The induction was carried when the cell confluence >80%. Medium containing 10 mM β-glycerophosphate, 50 μM vitamin C, and 100 nM dexamethasone was used to induce osteoblast differentiation. After 3-week induction, the osteoblasts differentiated from BMSCs were treated with α‐MEM (adding 10% FBS, a 1% antibiotic mixture of penicillin and streptomycin) with or without tetrandrine(1 μM) for 48 h. After the intervention, the cells were lysed to collect the proteins. Western blotting was used to detect the expression levels of RANKL and OPG in osteoblasts.

### Animal Experiments

Six‐week‐old female C57BL/6 mice were purchased from the Slack (Shanghai, China) and randomly divided into three groups (sham group, OVX group, and OVX + tetrandrine group, n = 6). All groups were anesthetized with 5% chloral hydrate and made small incisions on the dorsal skin and peritoneum, then the incision on the skin were closed with 5-0 nonabsorbable suture lines in sham group. Two ovaries and part of the oviduct of the mice in OVX group and OVX + tetrandrine group were removed. Pressed to stop any bleeding and closed the incision on the skin with 5-0 nonabsorbable suture lines. After the procedure, mice were allowed to recover for 48 h. From the third postoperative day, sham group and OVX group were intraperitoneal injected with normal saline containing 2% DMSO and OVX + tetrandrine group were intraperitoneal injected with 30 mg/kg of tetrandrine dissolved in normal saline with 2% DMSO. Mice in the sham group and OVX group were administered an equivalent volume of injected liquid. After 6 weeks of intervention, all animals were sacrificed. Blood samples were collected for ELISA tests while femur bone samples were collected for micro‐CT scanning and histological examination.

### Histological Examination

The femurs of mice were excised, fixed in 4% formaldehyde at room temperature and decalcified in 10% tetrasodium-EDTA aqueous solution for haematoxylin and eosin (H&E) and TRAP staining. Then, femur bone specimens were embedded by paraffin and sectioned to 4‐mm thickness with a microtome. Slides were stained with H&E and TRAP. Osteoclasts were visualized by TRAP staining. H&E stained sections were scanned by the Aperio Scanscope, and bone histomorphometric parameter, BV/TV, was analyzed by Image J software (NIH, Bethesda, MD, USA).

### Micro-CT Scanning

The femur was analyzed by microcomputed tomography (Skyscan, Antwerp, Belgium). The trabecular bone within the distal femur metaphysis was scanned at a 50 kV tube voltage and 500 μA current. Next, the three‐dimensional (3D) structure of the distal femur was reconstructed by Mimics 18.0 software (Materialise, Leuven, Belgium). Trabecular morphometry was characterized by measuring the bone volume per tissue volume (BV/TV), bone surface per tissue volume (BS/TV), bone mineral density (BMD), trabecular pattern factor (Tb.Pf), and trabecular number (Tb.N).

### Serum Biochemistry

Blood of the mice was collected *via* heart puncture before their sacrifice and sera were collected after centrifugation at 1,500 rpm for 20 min at room temperature. Serum levels of IL-6, TNF-a, TRAcp5b, osteocalcin (OCN), osteoprotegerin (OPG), RANKL, and C-telopeptide of type I collagen (CTX-I) were measured with ELISA kits. All the steps were operated according to the manufacturer’s instructions.

### Alkaline Phosphatase (ALP) Assay

BMSCs were seeded in 48-well plates at a density of 2×10^4^ cells/well. The induction was started when the cell confluence > 80%. Medium containing 10 mM β-glycerophosphate, 50 μM vitamin C, and 100 nM dexamethasone with different concentrations of tetrandrine (0, 0.25, 0.5, 1 μM) was used to induce osteoblast differentiation. The medium was changed every other day. After 1-week treatment, an ALP kit was used to stain osteoblasts. All operations were in accordance with the manufacturer’s protocol.

### Statistical Analysis

The experiments were performed at least three times. The data obtained are expressed as the mean ± standard error of the mean (SEM). Statistical analysis was performed using GraphPad Prism version 7.0 software (GraphPad Software, San Diego, CA, USA). Unpaired Student’s t test was used to compare 2 groups, and 1-way ANOVA was used to compare 3 or more groups. Probability values of P < 0.05 were considered statistically significant.

## Results and Discussion

### The Effect of Tetrandrine on RAW264.7 and BMM Cells Viability

**Figure 1:**
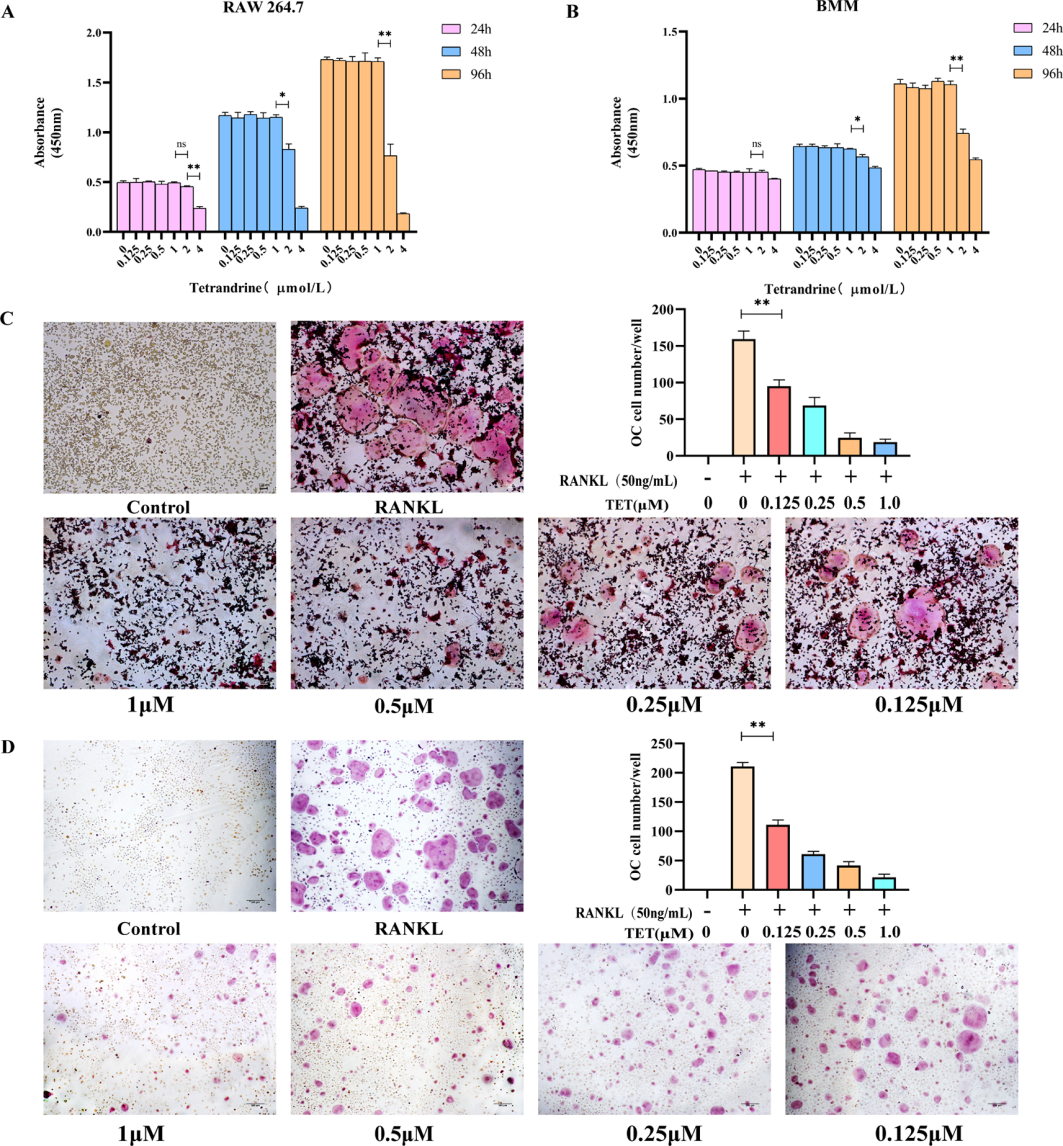
Tetrandrine inhibited osteoclastogenesis *in vitro*. **(A)** The influence of tetrandrine on cell growth in RAW264.7 cells at 24, 48, and 96 h. **(B)** The influence of tetrandrine on cell growth in RAW264.7 cells at 24, 48, and 96 h. **(C)** TRAP staining of RAW264.7 cells after treated with M-CSF (20 ng/ml), RANKL (50 ng/ml), and different concentrations of tetrandrine, then count the osteoclasts. **(D)** TRAP staining of BMMs after treated with M-CSF (20 ng/ml), RANKL (50 ng/ml), and different concentrations of tetrandrine, then count the osteoclasts. *P< 0.05, **P <0.01.

A CCK-8 assay was used to determine the cytotoxicity of tetrandrine (0.125, 0.25, 0.5, 1.0, 2.0, and 4 μm) on RAW264.7 and Bmms. the Absorbance At 450 nm Was Tested After 24, 48, and 72 H treatment and the data indicated that tetrandrine had no significant inhibitory effect on RAW264.7 and BMMS when the concentrations less than or equal to 1 μm (**Figures 1A**,**B**).

### Tetrandrine Inhibited RANKL-Induced Osteoclastogenesis *In Vitro*

To investigate the effects of tetrandrine on osteoclastogenesis *in vitro*, we used two kinds of standard models, RAW264.7 cells and BMMs. No TRAP-positive cells were found in the groups treated with M-CSF (20 ng/ml). The results indicated that M-CSF (20 ng/ml) cannot induce RAW264.7 or BMMS to osteoclasts without RANKL. For RAW 264.7 cells treated with tetrandrine (0, 0.125, 0.25, 0.5, or 1 μM), M-CSF (20 ng/ml) and RANKL (50 ng/ml), the number of TRAP-positive cells reduced in a dose-dependent manner compared with the control groups. Analysis of the numbers of TRAP-positive multinucleated cells are shown in **Figure 1C**. The same situation occurred in BMMs (**Figure 1D**). Together with the CCK-8 tests, these results showed that tetrandrine inhibited osteoclast formation in a dose-dependent manner and the inhibitation was not due to the loss of cell viability.

### Tetrandrine Inhibited RANKL‐Induced F‐Actin Ring Formation in Osteoclasts

**Figure 2:**
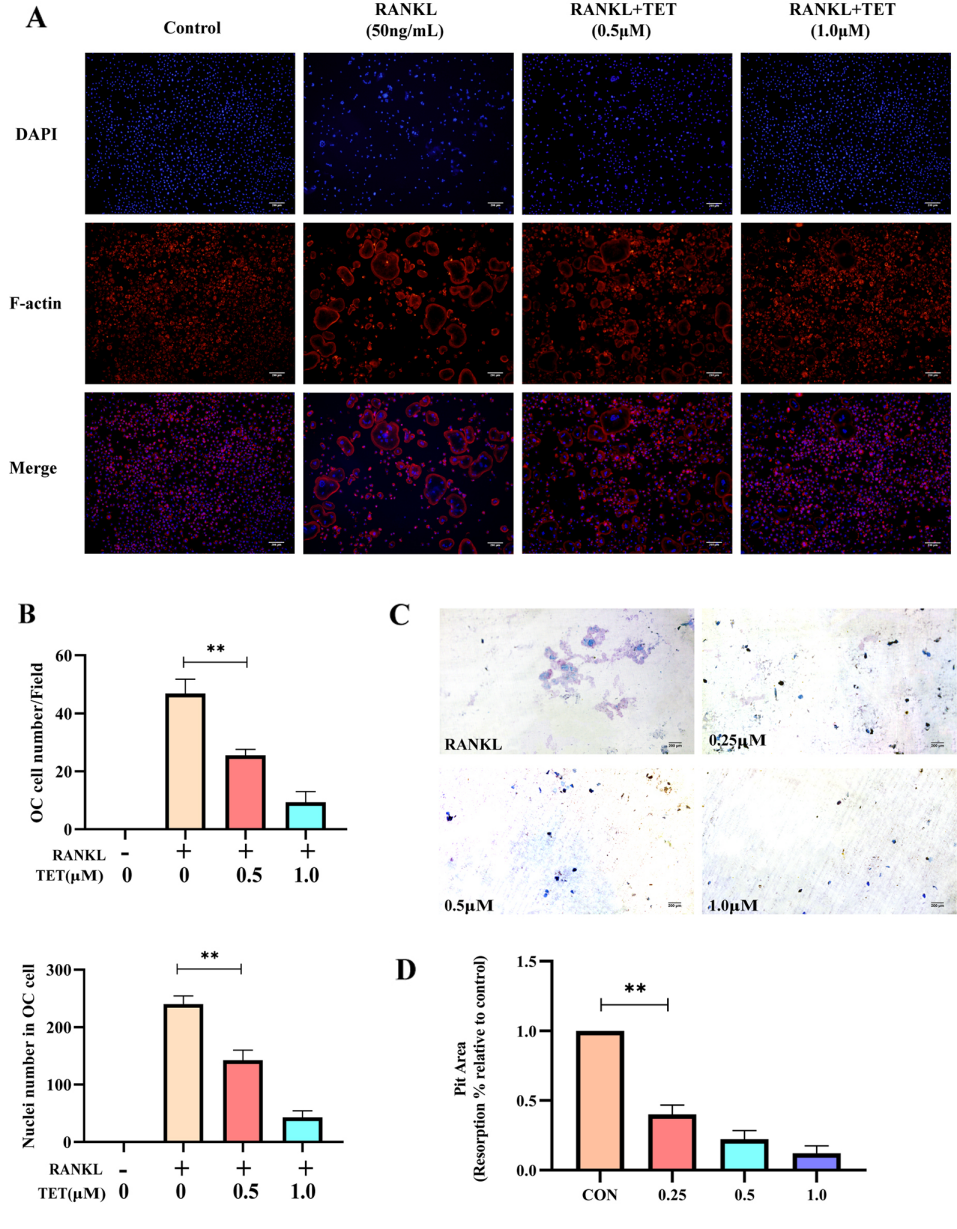
Tetrandrine inhibits osteoclast function *in vitro* **(A, B)** Tetrandrine suppresses RANKL-induced actin ring formation in BMMs. Osteoclasts having actin rings and nucleuses inside these osteoclasts were counted. **(C, D)**Tetrandrine inhibited the function of bone-resorption in bone marrow monocytes (BMMs). The slices were stained with toluidine blue to detect resorption pits and analyzed the picture by image J. **P < 0.01.

Actin ring formation is the most obvious feature of mature osteoclasts during osteoclastogenesis and a complete and large F‐actin structure is essential for osteoclasts involved in bone resorption (Takito et al., 2017). To determine the functions of osteoclasts, we examined whether osteoclast actin ring formation was affected by tetrandrine in RANKL-induced osteoclasts. The actin ring structure was detected when stained with rhodamine‐conjugated phalloidin. When the BMMs were treated with tetrandrine in different concentrations, the number of actin rings and nuclei number in osteoclasts were significantly reduced. The results were shown in **Figures 2A**,**B**, which indicated that tetrandrine reduced the formation of actin rings in mature osteoclasts in a dose-dependent manner.

### Tetrandrine Suppressed Bone Resorption by Osteoclasts

To further confirm whether tetrandrine suppressed the bone resorptive function of osteoclasts, bone slices were added to 48-well plates and mature osteoclasts were seeded onto them. Different concentration (0, 0,25, 0.5, and 1.0 μM) of tetrandrine, RANKL (50 ng/ml) and M-CSF (20 ng/ml) were added to the wells and the group treated with RANKL (50 ng/ml), M-CSF (20 ng/ml), and tetrandrine (0 μM) was used as the control group. After 48 h, the bone slices were stained with Toluidine Blue to detect resorption pits. Distinctly the resorption areas on the bone slices were smaller with the higher tetrandrine concentration (**Figure 2C**). The percentages of resorption pits on the surfaces of bone slices relative to control group were measured to evaluate bone resorptive function of osteoclasts. The results were shown in **Figure 2D**, indicating that tetrandrine inhabited the bone resorptive function of the mature osteoclasts.

### Tetrandrine Inhibits RANKL-Induced Activation of the MAPK, NF-KB, PI3K-AKT Pathway, and Ca2+ Oscillation

**Figure 3:**
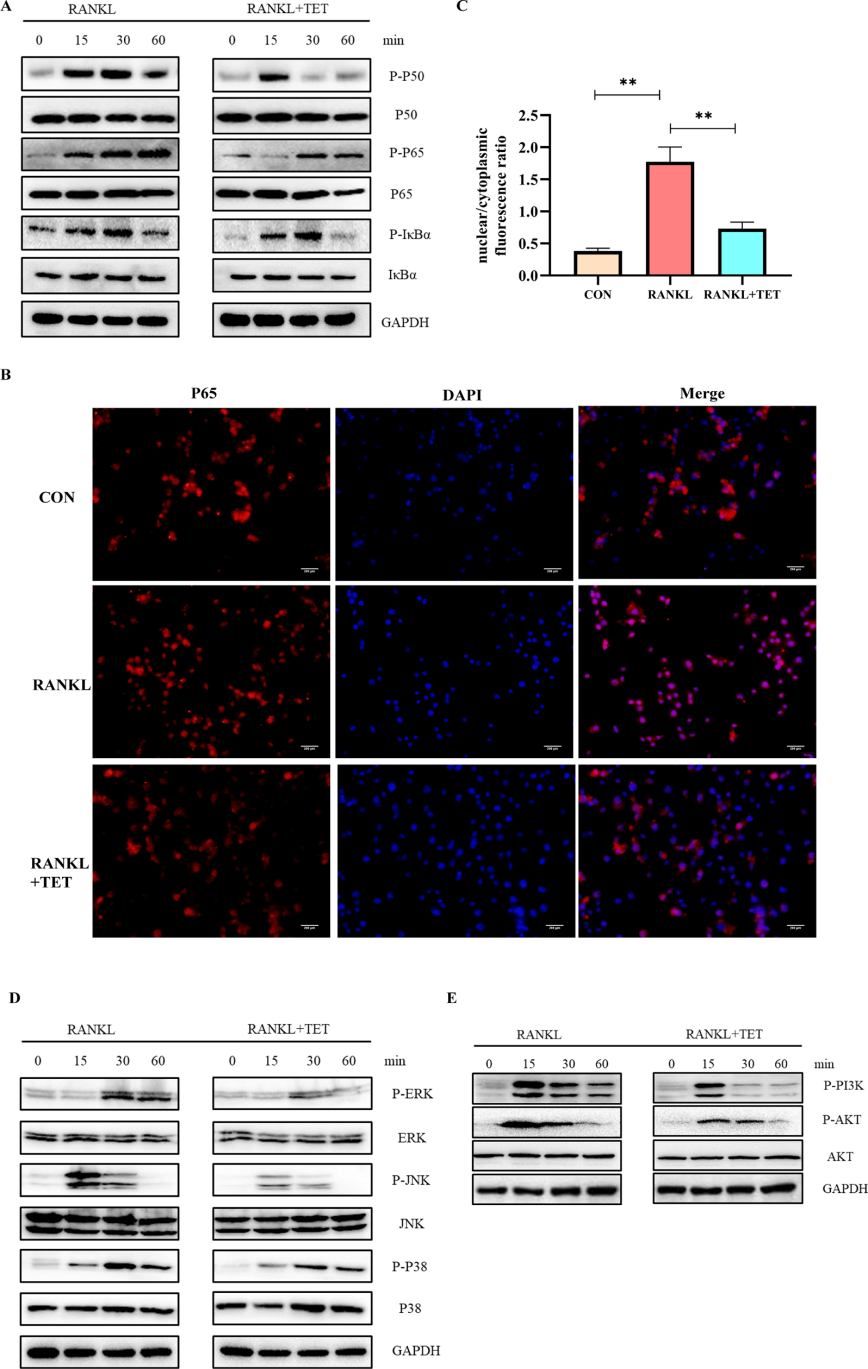
Tetrandrine inhibited RANKL-induced NF-kB, MAPK, and PI3K/AKT pathways activation. Bone marrow monocytes (BMMs) were treated with M-CSF (20 ng/ml), RANKL (50 ng/ml), and tetrandrine (1 μM) and lysed at 0, 15, 30, and 60 min. The proteins were collected to detect the levels of phosphorylation of key proteins by Western blotting. **(A)** Phosphorylation of key proteins in NF-kB pathways, including P50, P65, and IκBα, were detected by Western blotting. **(B)** P65 immunofluorescence images were captured by fluorescence microscopy to detect the inhibitory effect of tetrandrine on RANKL-induced P65 nuclear translocation. **(C)** The nuclear/cytoplasmic fluorescence ratios were detected by image J. **(D)** Phosphorylation of key proteins in MAPK pathways, including ERK1/2, JNK, and, P38, were detected by Western blotting. **(E)** Phosphorylation of key proteins in PI3K-AKT pathways, including PI3K and AKT, were detected by Western blotting. **P < 0.01.

**Figure 4:**
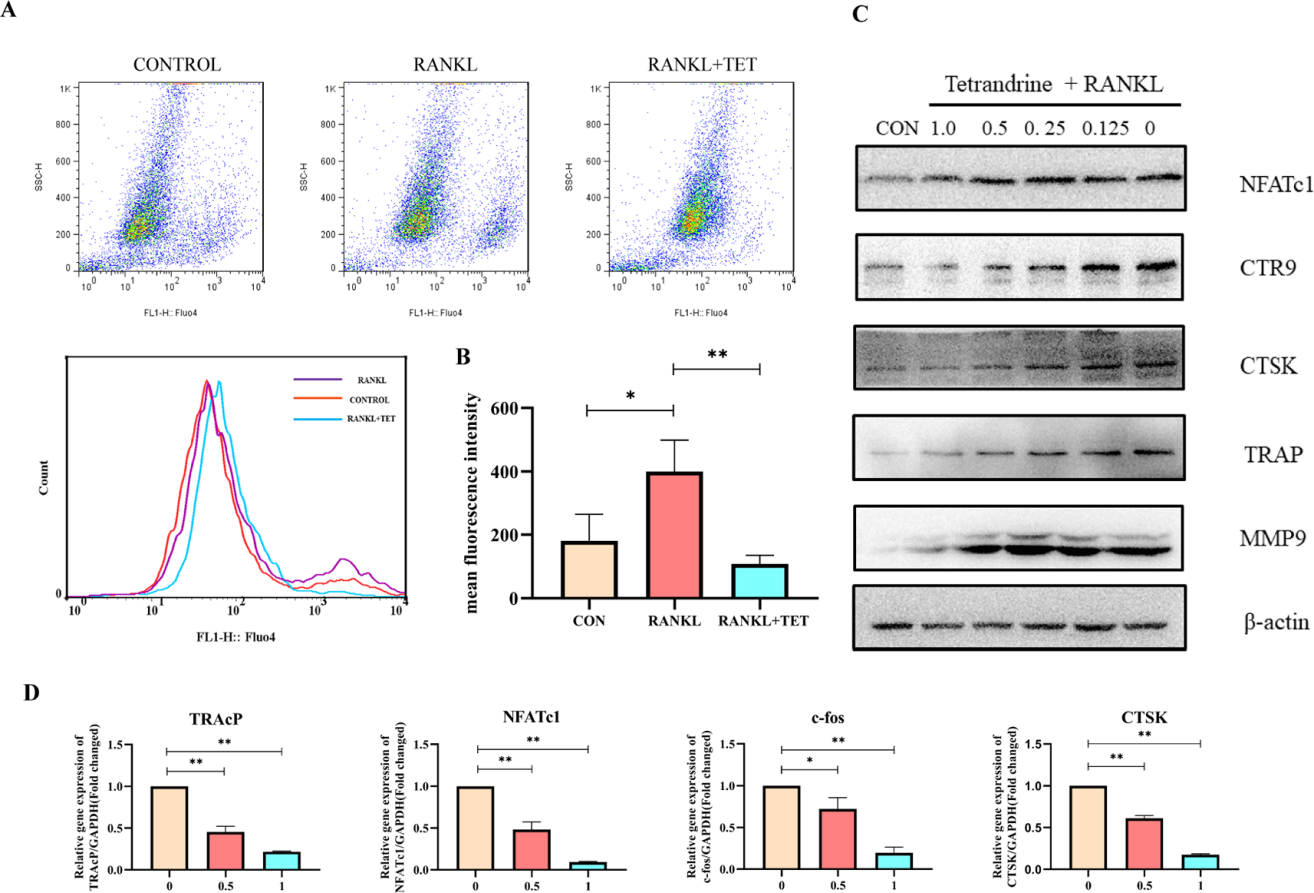
**(A)** Tetrandrine influenced the intracellular calcium oscillation caused by RANKL in bone marrow monocytes (BMMs). **(B)** Fluorescence intensity was detected by flow cytometry. The mean fluorescence intensity was analyzed by FlowJo. **(C)** RAW264.7 cells were harvested after induced by RANKL (50 ng/ml) and tetrandrine (0, 0.125, 0.25, 0.5, and 1 μM) for 5 days. The proteins were used to detect osteoclastogenic proteins levels of NFATc1, CTR9, CTSK, TRAP, and MMP9. **(D)** Tetrandrine down‐regulates osteoclastogenic gene expression of *NFATc1, TRAP, CTSK*, and *c-fos*. RAW264.7 cells were stimulated with RANKL and cultured with different concentrations of tetrandrine. RNA was extracted after 5 days. A qPCR analysis was carried to detect osteoclastogenic gene expression. Data were analyzed using the 2−ΔΔCT method. The specific primers used are listed in **Table 1**. *P < 0.05, **P < 0.01.

RANKL-induced activation of MAPK, NF-kB, PI3K-AKT pathways, and Ca^2+^ oscillation is necessary for osteoclast differentiation (Feng and Teitelbaum, 2013). Cells were treated as description in methods and then evaluated by Western blotting at 0, 15, 30, and 60 min to observe phosphorylation of the proteins. Western blot assays were used to determine that tetrandrine could inhibit RANKL-induced phosphorylation and degradation of IκBα, P50, and P65. The results illustrated that tetrandrine inhibits the phosphorylation of NF-kB pathway (**Figure 3A**). To further determine whether tetrandrine inhibits NF-kB pathway, immunofluorescence staining of P65 with or without tetrandrine was performed in BMMs. The results showed that P65 was phosphorylated and translocated to the nucleus when treated with RANKL. Compared to RANKL treated group, the nuclear translocation of P65 was blocked in tetrandrine treated group with the same concentration of RANKL (**Figures 3B**,**C**). In MAPK pathway, we examined the phosphorylation of ERK1/2, JNK, and P38 by Western blotting, the results showed that the phosphorylation of this proteins were obviously decreased after treated with tetrandrine (**Figure 3D**), which indicated that tetrandrine inhibited MAPK pathway in osteoclastogenesis. In AKT pathway, we examined the phosphorylation of PI3Kand AKT by Western blot analysis. The inhibition of phosphorylated PI3K and phosphorylated AKT when treated with tetrandrine showed that tetrandrine can inhibit RANKL-induced activation of AKT pathway in osteoclasts (**Figure 3E**). Additionally, we found that tetrandrine significantly suppressed RANKL‐induced Ca^2+^ oscillation in BMMs (**Figures 4A**,**B**), which indicated that inhibitory effect on calcium signaling was part of the mechanism in which tetrandrine inhibited osteoclastogenesis. Taken together, tetrandrine inhibited MAPK, NF-kB, PI3K-AKT pathways, and Ca^2+^ oscillation in the osteoclastogenesis of BMMs.

### Tetrandrine Down‐Regulates Osteoclastogenic Gene Expression

We next examined the changes on osteoclastogenic gene expression caused by tetrandrine with real‐time PCR analysis. It is known that osteoclast differentiation depends on the expression of a lot of related marker genes, such as *NFATc1, TRAcP, c-Fos*, and *cathepsin K*, among which *NFATc1* is one of the most well-known master regulator of osteoclastogenesis and function (Nakashima et al., 2012). In this experiment, RAW 264.7 cells were treated with RANKL for 5 days with or without different concentrations of tetrandrine. The upregulation of *c-Fos, cathepsin K, TRAcP*, and *NFATc1* gene expression was inhibited markedly by tetrandrine in a dose‐dependent manner (**Figure 4D**). At the concentration of 1.0 μM, tetrandrine obviously inhibited the expression of these genes. Meanwhile, we examined the protein levels of MMP9, CTSK, CTR9, TRAP, and NFATc1. The results showed that tetrandrine inhibited the expression of these proteins in a dose‐dependent manner (**Figure 4C**). The PCR analysis and Western blotting results showed that tetrandrine inhibited osteoclast specific genes expression and decreased osteoclast specific proteins level, and blocked the osteoclastogenesis of RAW264.7 cells.

### Effect of Tetrandrine on Bone Loss in OVX Mice

**Figure 5:**
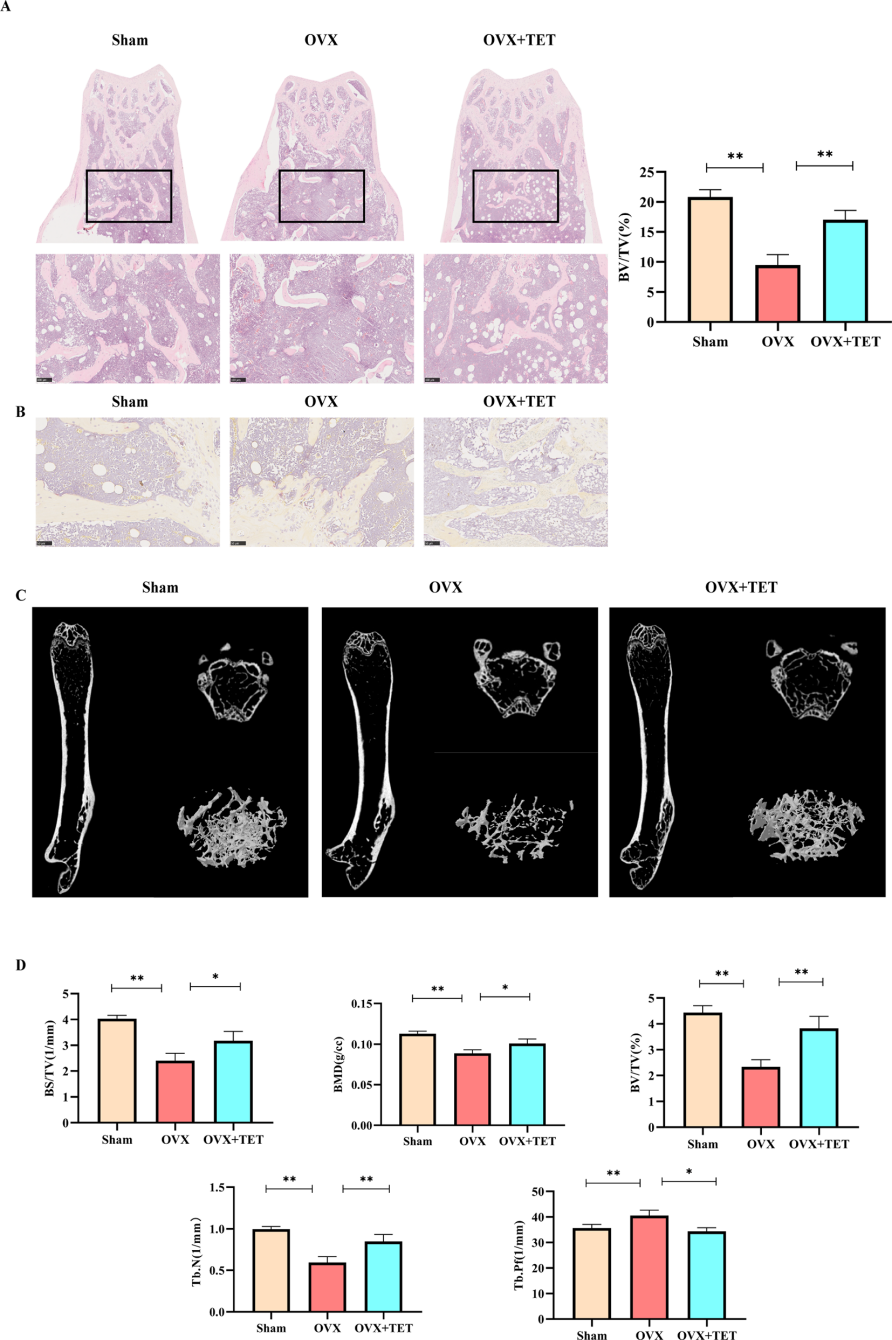
Tetrandrine inhibited ovariectomy-induced bone loss and reduced osteoclasts *in vivo.* Three groups were sham group (Sham), ovariectomized group (OVX), and ovariectomized group with drug injection group (OVX+TET). **(A)** Representative H&E staining of femoral sections. BV/TV was analyzed by image J. **(B)** Representative TRAP staining of femoral sections. **(C)** Representative micro-computed tomography sections of femur. BV/TV, BS/TV, BMD, Tb. Pf, Tb. N were analyzed by GraphPad. The differences between Sham, OVX, and OVX+TET groups were shown in the charts. **(D)** The serum levels of IL-6, TNF-a, TRAcp5B, and OCN were examined. *P < 0.05, **P < 0.01.

**Figure 6:**
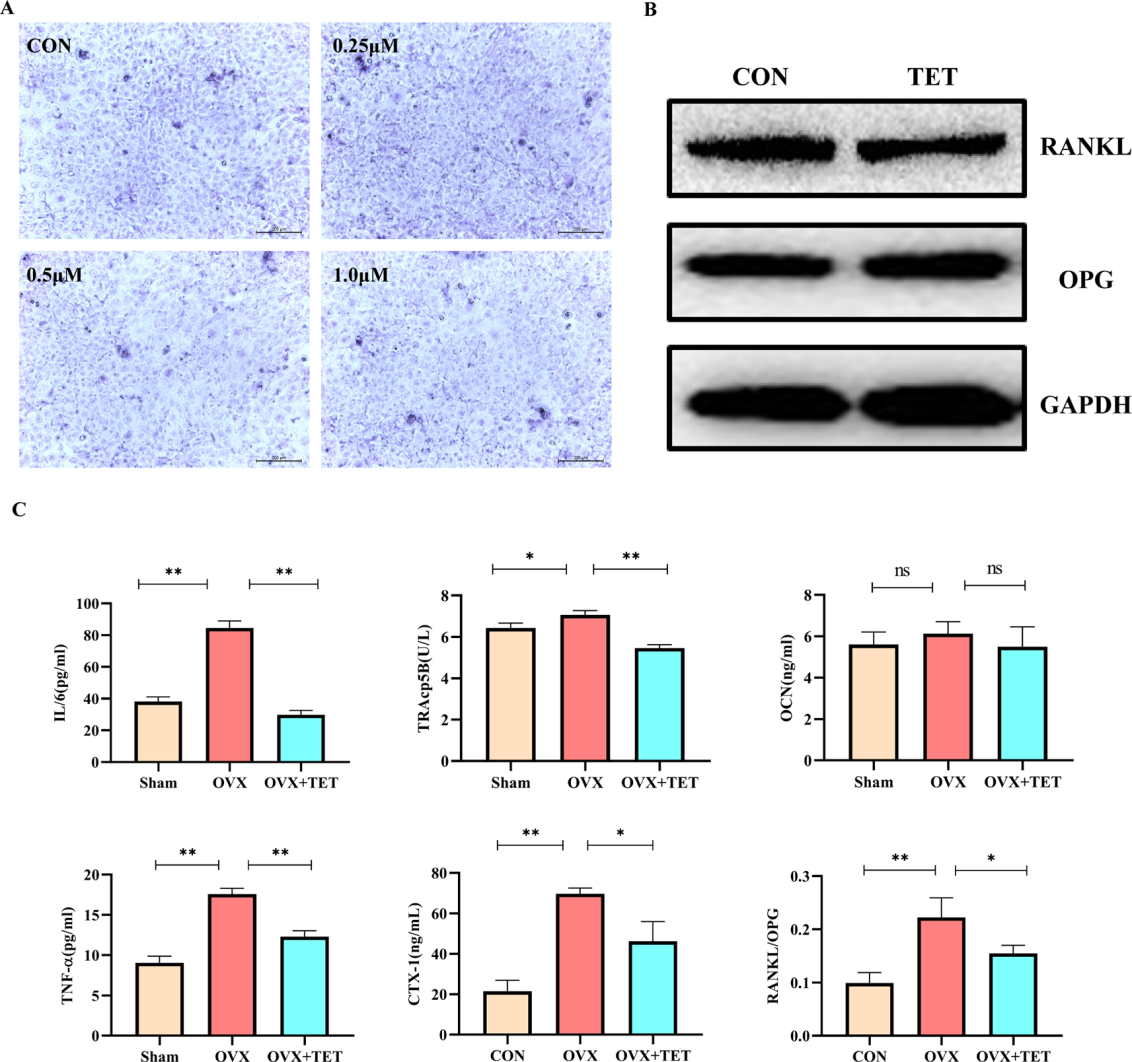
**(A)** Bone marrow stromal cells (BMSCs) were induced by medium containing 10 mM β-glycerophosphate, 50 μM vitamin C, and 100 nM dexamethasone with different concentrations of tetrandrine (0, 0.25, 0.5, and 1 μM). After 1-week induction, the cells were stained by an alkaline phosphatase (ALP) kit. **(B)** BMSCs were induced by medium containing 10 mM β-glycerophosphate, 50 μM vitamin C, and 100 nM dexamethasone with or without tetrandrine (1 μM). After 3-week induction, the cells were lysed to collect the proteins. The OPG and RANKL levels were detected by Western blotting. **(C)** The serum levels of IL-6, TNF-a, TRAcp5B, CTX-1, OCN, and RANKL/OPG were examined. The differences between Sham, OVX, and OVX+TET groups were shown in the charts. *P < 0.05, **P < 0.01. ns, no significance.

We built an OVX mouse model to mimic PMOP for the purpose of evaluating the effects of tetrandrine on bone loss and examining the potential therapeutic benefits of tetrandrine on bone loss *in vivo*. We carried out H&E stain and microcomputed tomography of trabecular bone within the distal femur metaphysis to evaluate whether tetrandrine inhibited the bone loss in OVX. Then, we carried out TRAP stain and ELISA test to further determine whether tetrandrine inhibited the bone loss through inhibiting osteoclastogenesis rather than promote osteogenesis *in vivo*. According to the results of H&E staining, the bone volume per tissue volume (BV/TV) of mice were markedly increaed in tetrandrine treated group compared with OVX group (**Figure 5A**). The result of microcomputed tomography showed that the mice in OVX + tetrandrine group had higher BMD, BS/TV, bone mineral density (BMD), trabecular pattern factor (Tb.Pf), and trabecular number (Tb.N) compared with OVX mice treated with normal saline including 2% DMSO (**Figures 5C**,**D**). These results indicated that the bone loss in OVX mice was alleviated when treated with tetrandrine (30 mg/kg) every other day. In the TRAP assay, TRAP-positive cells were decreased in drug-treated mice compared with OVX mice (**Figure 5B**), indicating that osteoclasts are inhibited in drug-treated group. Meanwhile, in ELISA assay, the levels of CTX-1 and TRAcp5B in serum were significantly lower in drug treated mice than OVX mice, which indicated that the viability of osteoclast *in vivo* was significantly decreased. However, the level of OCN, an indicator of osteogenesis (Han et al., 2018), had no obvious difference(**Figure 6C**), which indicated that the level of osteogenesis in the body has no significant change. These results illustrated that tetrandrine inhibited osteoclastogenesis rather than promoted osteogenesis *in vivo*. Based on previous studies, tetrandrine inhibits the activation of T lymphocyte, which secrets large amounts of IL-6, TNF-β, and is one of the most important sources of RANKL *in vivo* (Ho et al., 2004; Takayanagi, 2007; Weitzmann and Ofotokun, 2016). To investigate whether tetrandrine inhibited bone loss not only by blocking osteoclastogenesis but also by down-regulating the serum levels of IL-6, TNF-β, and RANKL/OPG, we carried the ELISA tests of IL-6, TNF-β, and RANKL/OPG. The results showed that the serum levels of IL-6, TNF-β, and RANKL/OPG were highly decreased after treated with tetrandrine (**Figure 6C**), indicating that tetrandrine’s inhibitory function of bone loss may partly due to its inhibitory effects on T cell activation, proliferation, and cytokine expression.

### Effect of Tetrandrine on Osteogenesis

As shown in **Figure 6A**, when stained by an ALP kit, there is no significant difference between the groups with or without tetrandrine after one-week intervention. The results indicated that tetrandrine had no significant inhibitory effect on the survival and differentiation of BMSCs at the concentration below 1 μM. As shown in **Figure 6B**, the protein expressions of OPG and RANKL in osteoclasts after treated with tetrandrine (1 μM) had no obvious change, compared with control group. Combined with the previous results, we can deduce that the decreases of RANKL and OPG levels *in vivo* may due to the inhibitory effect of tetrandrine on activated T lymphocytes.

## Discussion

**Figure 7:**
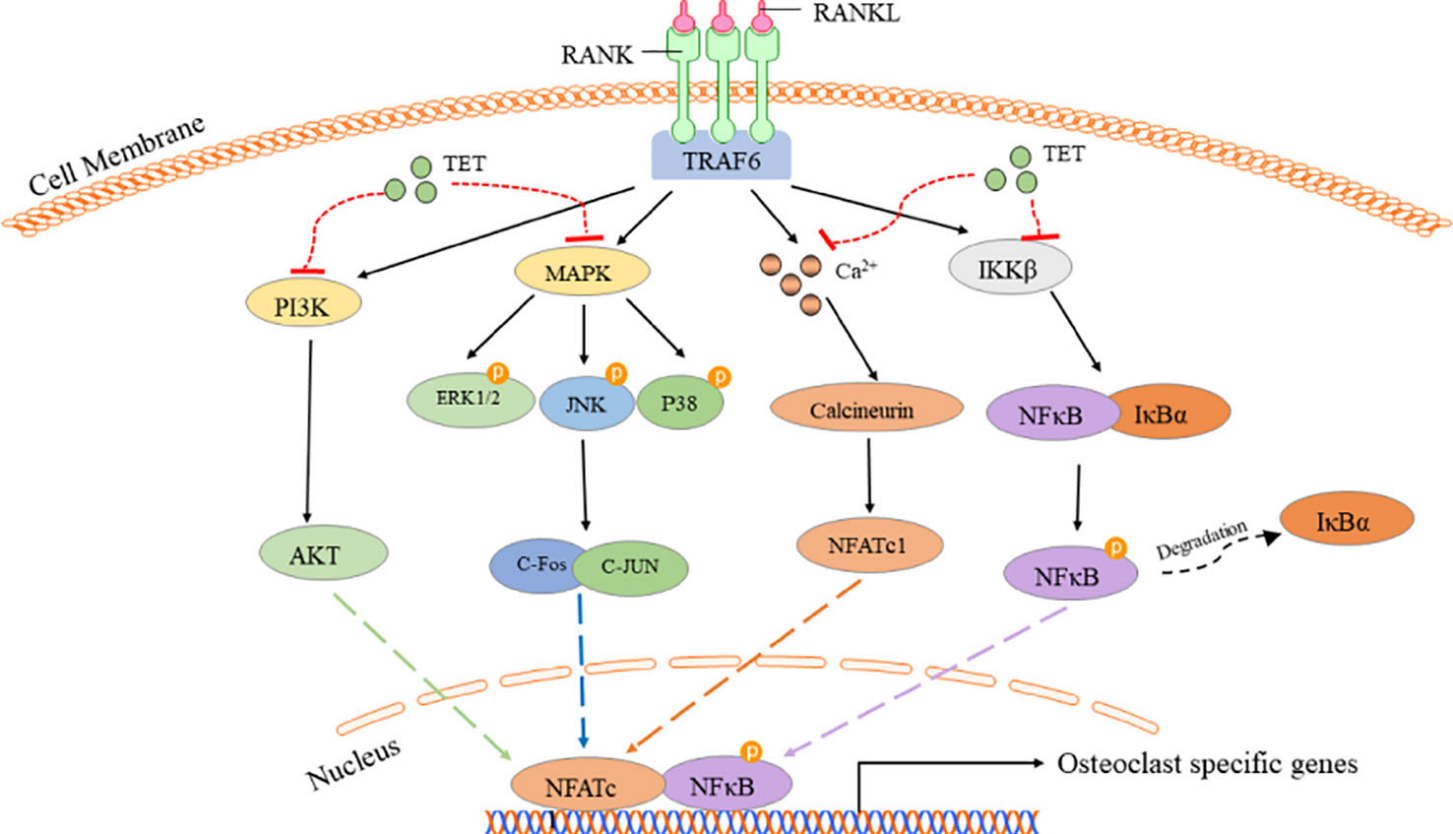
Tetrandrine inhibited RANKL-induced osteocalstogenesis through multiple pathways.

In this study, we found that tetrandrine markedly prevented bone loss *in vitro* and inhibited osteoclastogenesis *in vivo*. Previous studies focused more on the role of tetrandrine in RA (Jia et al., 2019). For the first time, we verified the effects of tetrandrine on osteoporosis in OVX mice model. Mechanically, tetrandrine inhibited the RANKL‐induced differentiation and function of osteoclasts and inhibiting the activation of the NF-kB, MAPK, Ca^2+^, and PI3K/AKT signaling pathways (**Figure 7**). As tetrandrine had no obvious effect on osteoblast and had a significant effect on attenuating bone loss *in vivo*, it further indicated that tetrandrine can be used as a treatment for patients with PMOP, especially the patients with both PMOP and RA.

As the most common type of primary osteoporosis, PMOP is resulted from many reasons, among which estrogen withdrawal plays a key role (Watts, 2014; Farr and Khosla, 2015; Black and Rosen, 2016). Estrogen withdrawal brings about T cell activation, proliferation, and cytokine expression, further leads to increased formation and activation of osteoclast (Manolagas, 2010; Khosla et al., 2012; Karsdal et al., 2014). Osteoclast is a kind of multinucleated giant cells which had the function of bone resorption. Overactivation of osteoclast leaded to a series of bone destruction-related diseases, such as autoimmune arthritis, periodontitis, postmenopausal osteoporosis, Paget’s disease, and bone tumors. Therefore, inhibition of osteoclast formation and/or its function became a promising strategy for treating pathological bone loss. Although many drugs are used to treat PMOP clinically and had good effects, many side effects showed up after long-term treatment. For example, bisphosphonates, the most popular drug used in clinical, may suppress bone formation and lead to post-fracture nonunion after long-term treatment (Reid, 2015). Moreover, bisphosphonates had no obvious effects on RA, which occurred with PMOP simultaneously in a large proportion of cases. Therefore, agents with less side effects and more indications are required.

Many monomers extracted from traditional Chinese medicine have inhibitory effects on osteoclastogenesis (Chen et al., 2017). Tetrandrine, a bis-benzylisoquinoline alkaloid isolated from Stephania tetrandra S Moore and other Chinese herbs, has been shown to have anti-inflammatory, antineoplastic, immunologic, and antiallergenic effects (Mei et al., 2015; Liu et al., 2017; Yu et al., 2018; Jia et al., 2019). Clinically, it has been widely used in the treatment of rheumatoid arthritis. Previous study had shown that tetrandrine relieved the bone resorption in rheumatoid arthritis by inhibiting osteclastogenesis (Jia et al., 2019). However, whether tetrandrine can be used to treat PMOP stayed unknown. Therefore, we carried out this study to explore the effects of tetrandrine on PMOP as well as the underlying molecular mechanisms. *In vivo*, the results of H&E staining, TRAP staining together with microcomputed tomography suggested that tetrandrine markedly reduced the number of activated osteoclasts around the trabecula and attenuated bone loss in OVX mice. In serum, the levels of CTX-1, and TRAcp5B significantly lower in tetrandrine treated OVX mice, while OCN had no obvious difference. *In vitro*, the results of TRAP staining, F-actin ring formation and bone pit assay indicated that tetrandrine significantly inhibited osteoclast differentiation and its function in RAW 264.7 cells and BMM cells. Therefore, we considered that tetrandrine attenuated bone loss in PMOP by inhibiting osteoclastogenesis. According to previous studies, T lymphocytes are overactivated and secrete many cytokines after the withdrawal of estrogen. Activated T lymphocytes then secret large amounts of IL-6, TNF-β, and become one of the most important sources of RANKL *in vivo* (Takayanagi, 2007). It is reported that tetrandrine inhibits the activation of T lymphocyte (Ho et al., 2004). We found that the serum levels of IL-6, TNF-β, and RANKL/OPG were highly decreased after treated with tetrandrine and these results indicated that tetrandrine’s inhibitory function of bone loss may partly due to its inhibitory effects on T cell activation, proliferation, and cytokine expression.

Osteoclastogenesis is regulated by numerous factors, among which RANKL may be the most important one (Croft et al., 2017; Cao, 2018; Ahern et al., 2018). A large series of downstream signaling cascades were activated after RANKL engages with RANK, among which NF-kB, MAPK, Ca^2+^, and PI3K/AKT signaling play major roles in signal transduction. The NF-kB family includes p105 (NF-kB1), p100 (NF-kB2), RelA (P65), RelB, and Relc. After P105 is converted to P65 and forms a dimer with P50, IκBα is subsequently degraded in the cytoplasm. Then, the dimer is translocated to the nucleus and binds to specific DNA sites to regulate osteoclastogenesis (Krum et al., 2010). In this study, we found that tetrandrine prevented RANKL-induced translocation of P65 from cytoplasm to nucleus by fluorescence in BMM cells. Meanwhile the results of Western blot showed that the phosphorylation of P50, P65, and IκBα were suppressed in drug treated group compared with the RANKL treated group, which together with fluorescence indicated that tetrandrine could block the canonical NF-kB pathway. PI3K/AKT is another important signaling pathway in osteoclastogenesis (Liu et al., 2016). RANK activates Src family kinase signaling, which leads to AKT activation through interactions between TRAF6 and Cbl scaffolding proteins. Additionally, Ca^2+^ oscillation is the downstream of TRAF6, and then leads to nuclear translocation and activates NFATc1 (Nemeth et al., 2011). In this study, activation of Ca^2+^ oscillation was abrogated when treated with tetrandrine, indicating tetrandrine inhibited Ca^2+^signaling pathway in osteoclastogenesis. RANKL‐induced osteoclastogenic marker expression was also tested by PCR. The expression of c-Fos, NFATc1, TRAcP, and cathepsin K were obviously decreased when treat with tetrandrine in RAW.264.7 cells. Taken together, tetrandrine inhibited osteoclastogenesis through NF-kB, Ca^2+^, and PI3K/AKT signaling pathways, which significantly suppress RANKL‐induced osteoclastogenic marker expression.

Considering many limitations in our study, further investigations are needed to supplement our research. First of all, tetrandrine, supposed widely used in clinical for PMOP, has many disadvantages, such as high toxicity, poor water solubility, and low targeting efficiency. More superior derivatives are needed to improve these disadvantages. Second, tetrandrine inhibits three major RANKL-induced conventional signaling pathways, indicating that it may have an effect on upstream molecule. A DARTS (Drug Affinity Responsive Target Stability) assay could be carried out to look for the drug target of tetrandrine. Finally, extensive clinical application of tetrandrine makes it possible to do some clinical studies on tetrandrine. In this situation, we may have a more profound understanding of the role of tetrandrine in PMOP.

In conclusion, according to the *in vivo* and *in vitro* results in our study, tetrandrine, as a novel inhibitor of osteoclastogenesis by suppressing NF-kB, MAPK, Ca^2+^, and PI3K/AKT signaling pathways, is suggested to be a potential therapeutic candidate in treating PMOP.

## Data Availability Statement

The raw data supporting the conclusions of this article will be made available by the authors, without undue reservation, to any qualified researcher.

## Ethics Statement

The animal research was reviewed and approved by the Shanghai Pudong Hospital Animal Ethics Committee.

## Author Contributions

ZZ, ZQ, and BY: study design. DL and XZ: data collection. SN and FC: data analysis. XZ: data interpretation. ZZ and ZQ: drafting manuscript. ZK, FZ, and SN: revising manuscript content. BY: approving final version of manuscript.

## Conflict of Interest

The authors declare that the research was conducted in the absence of any commercial or financial relationships that could be construed as a potential conflict of interest.
